# Primary care nurses’ performance in motivational interviewing: a quantitative descriptive study

**DOI:** 10.1186/s12875-015-0304-z

**Published:** 2015-07-25

**Authors:** Ann-Sofi Östlund, Marja-Leena Kristofferzon, Elisabeth Häggström, Barbro Wadensten

**Affiliations:** Department of Public Health and Caring Sciences, Uppsala University, BMC, Box 564, Uppsala, 751 22 Sweden; Faculty of Health and Occupational Studies, University of Gävle, Gävle, 801 76 Sweden

**Keywords:** Self-ratings, Motivational interviewing, Motivational interviewing treatment integrity code, Nurse, Performance, Primary care, Proficiency/competency

## Abstract

**Background:**

Motivational interviewing is a collaborative conversational style intended to strengthen motivation to change. It has been shown to be effective in addressing many different lifestyle problems as well as in chronic disease management, and many disease prevention guidelines promote use of motivational interviewing. The aim of the present study was twofold: to assess to what extent the primary care nurses in the study perform motivational interviewing according to the Motivational Interviewing Treatment Integrity Code and to investigate how the participating primary care nurses rated their own performance in motivational interviewing.

**Method:**

The study was based on twelve primary care nurses’ audio-recorded motivational interviewing sessions with patients (total 32 sessions). After each session, the nurses completed a questionnaire regarding their experience of their own performance in motivational interviewing. The audio-recorded sessions were analyzed using Motivational Interviewing Integrity Code 3.1.1.

**Results:**

None of the nurses achieved beginning proficiency in all parts of any motivational interviewing sessions and two nurses did not achieve beginning proficiency in any parts or sessions. Making more complex than simple reflections was the specific verbal behavior/summary score that most nurses achieved. Beginning proficiency/competency in “percent open questions” was the summary score that fewest achieved.

**Conclusion:**

Primary care nurses did not achieve beginning proficiency/competency in all aspects of motivational interviewing in their recorded sessions with patients, where lifestyle change was discussed. This indicates a need for improvement and thus additional training, feedback and supervision in clinical practice with motivational interviewing.

## Background

The shift from acute to chronic health problems has placed new demands on healthcare systems [1]. If shared risk factors could be reduced and/or eliminated through more effective preventive healthcare, most morbidity and premature mortality due to chronic disease would be preventable [2]. It is therefore necessary to invest in human resources to produce health interventions of high quality. Such investments must ensure that the right categories and numbers of healthcare staff, such as primary care nurses, are trained and maintain their productivity, quality and competency [1].

Motivational interviewing (MI) is a collaborative conversational style used to strengthen a person’s own motivation and commitment to change [3]. It has been shown to be effective in addressing many different lifestyle problems and in chronic disease management [4–7]. In Sweden, training in and use of MI has grown ever since the Swedish Government first initiated it in 2004 [8–10]. Thanks to guideline recommendations [11], training in MI is now offered to all categories of healthcare staff. Courses in MI typically include a theoretical part covering the method and a practical part covering the techniques; they usually last for two to four days [12, 13] and are arranged by county councils or private educators. MI training is also offered as a special university course up to ten weeks in duration or as part of university courses offering, for example, continued education for district nurses, diabetes nurses or asthma nurses and courses on disease prevention.

MI involves shaping a conversation with a patient so that, in the end, the patient has talked him-/herself into a change that is in his/her own best interest. It can also be conceived of as a way of being with people/patients. The so-called spirit of MI is the underlying perspective with which the user of MI enters into the practice of MI. The four key elements of the spirit of MI are: ***partnership*** – which concerns active collaboration between experts; ***acceptance*** - which involves respecting all humans’ *absolute worth*, showing *accurate empathy*, supporting *autonomy* and *affirming* patients’ effort and strength; ***compassion*** - which means prioritizing and promoting patients’ needs and welfare; ***evocation*** – which concerns eliciting patents’ own motivation and resources. To practice MI, five core skills are required: ***Asking open questions*** to explore patients’ own references, reasons and prerequisites for change, to create collaboration and to evoke motivation. ***Affirming*** to acknowledge and comment on patients’ positive intentions, abilities and efforts. ***Reflecting*** to give the patient an opportunity to hear once again what he/she has said, as well as to give the provider (e.g., nurse, physician, therapist) a chance to surmise what the patient meant in order to encourage the patient to keep deliberating and exploring. ***Summarizing*** to promote understanding and show the patient that he/she is being listened to and that what he/she has to say is of value. Providing ***information and advice*** with permission or when the patient asks for them [3].

Validated instruments have been developed to give MI providers feedback. Such feedback can be used to improve their skills in and use of MI as well as to ensure good patient outcomes [14–17]. Validated instruments are also recommended for measuring adherence to MI prior to evaluation of outcomes [14, 18]. The Motivational Interviewing Treatment Code 3.1.1 (MITI) is one of the instruments developed to test providers’ MI skills [19] and has previously been used in Swedish healthcare settings [20, 21]. There is also a more recent, updated version of the MITI, version 4.1 that is not yet in use in Swedish settings. According to the MITI 3.1.1 manual [19], coders generally need to participate in more than 40 h of initial training to achieve good inter-rater reliability in MITI; in addition, they probably require weekly group training in coding. The coding should preferably cover the first twenty minutes of a recorded MI conversation between the MI provider and client/patient. The coding involves two different processes: global estimations and frequency calculation/counts of specific behaviors. It is also possible to calculate a summary score to determine the MI provider’s MI competence (beginning proficiency (approved beginners) or competency (competent) levels). The coders need to know the specific target behavior for the recorded MI conversation, as such knowledge allows them to more accurately judge whether the MI provider is directing the session toward the behavior target [19]. Since development of the MITI, studies have used the instrument for different purposes and settings. It has been used particularly in addiction treatment studies [22, 23], MI training interventions and to evaluate clinical use of MI in different settings and on different professions [24–30], including nurses [25, 31–33] and has shown varying results, from achieved proficiency in all parts/variables of the MITI to lack of proficiency across the board. A few studies [16, 20, 21, 34–36] have used the MITI in primary care settings, three of which [16, 28, 30] used data from a project on physicians called CHAT, where only some of the physicians had training in MI. Both studies by Bohman et al. [20] and Efraimsson et al. [21] were performed in Swedish primary care with nurses: one in the child health services [20], the other at chronic obstructive pulmonary disease clinics [21]. Bohman et al. [20] aimed at evaluating an enhanced MI training program and Efraimsson et al. [21] at describing to what extent nurses used MI in smoking session after a few days of prior training in MI. None of the nurses in the two studies [20, 21] achieved beginning proficiency in MI. In the study by Efraimsson et al. [21], six nurses conducted 26 smoking cessation sessions based on MI with 13 patients. The participating nurses most commonly provided information followed by asking closed questions and making simple reflections. They seldom asked open questions, evocated motivation and/or collaborated with the patient. One nurse achieved the beginning proficiency level on one of the MITI variables.

There are certain challenges associated with learning and implementing MI. For this reason, there has been a call in the literature to evaluate MI users’ expertise and to examine whether MI has actually been learned, put into practice and used properly [37, 38]. Despite the growing need for effective methods to help patients make lifestyle changes, and despite the costs of MI training and implementation, according to a survey study in a Swedish primary care setting, less than half of nurses trained in MI actually used it [39]. It is therefore crucial to further investigate adherence to MI among primary care nurses trained in the method. Because use of MI is relatively new in Sweden, to the best of our knowledge, only one previous Swedish study [21] has examined use of MI among nurses in primary care in natural settings and following earlier training in MI.

### Aims

The aim of the present study was twofold: to assess to what extent the primary care nurses in the study perform MI according to the MITI and to investigate how the participating primary care nurses rated their own MI performance.

### Research questions

1. How do primary care nurses score on MI performance with regard to the five global dimensions of MITI (Evocation, Collaboration, Autonomy-Support, Direction and Empathy) and how do they rate their own performance on these dimensions?
2. How many times during an MI session do specific MITI-defined behaviors occur (giving information, asking open and closed questions, making simple and complex reflections) and how do primary care nurses rate their own performance regarding these specific behaviors?
3. To what extent do primary care nurses, in their recorded sessions, achieve beginning proficiency/competency in MI as stipulated by established MITI thresholds?

## Methods

### Design

The present study was quantitative and descriptive in design. The purpose of a descriptive study is to examine, observe and describe a situation/sample/variable as it naturally occurs [40, 41], without investigator interference [40].

### Sample

**Fig. 1:**
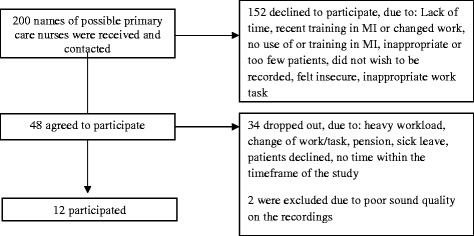
Recruiting/Sampling process and dropout rate

The inclusion criteria were that participants should be primary care nurses working in somatic primary care who had some kind of training in MI and who reported using the method in their work. The primary care nurses were recruited from primary care centers in two county council districts in central Sweden. The two county council districts were chosen with the express purpose of obtaining a varied and large sample of primary care nurses from different settings. In these two districts, there was a large university town, several larger and smaller towns as well as rural areas with small communities. The selection process is demonstrated in Fig. 1. Each participating nurse was asked to audio-record three sessions with patients. In the end, twelve primary care nurses participated in the study. They were all women, between 43-62 years (mean 54.6 years) and had worked as nurses for 15-40 years (mean 28.1 years); they will henceforward be referred to as nurses. The majority of the nurses had received their training in MI from the county council. Four nurses had received feedback on their own MI sessions during additional MI training (not offered by the county council). The characteristics of the patients participating in the sessions are presented in Table 1.

**Table 1:** Characteristics of the patients

Characteristics	
Age (years), range (mean)	23-76 (51.5)
Gender, *n* (%)	
*Female*	21 (65.6)
*Male*	11 (34.4)
Area of residence, *n* (%)	
*Urban area*	20 (62.5)
*Rural area*	12 (37.5)
Education, *n* (%)	
*Compulsory school*	10 (31.3)
*High school degree*	14 (43.8)
*University degree*	8 (25.1)
Occupation, *n* (%)	
*Employed*	21 (65.6)
*Retired*	9 (28.1)
*Studying*	1 (3.1)
*Unemployed*	1 (3.1)

### Data collection

#### Audio-record

The data were collected through audio-recorded MI sessions between a nurse and patient. The nurses were asked to audio-record MI sessions regarding some change in a patient’s lifestyle/habits/behavior. Each participating nurse was asked to select and record sessions from her normal, planned patient visits, as they occurred in her normal work settings. They contributed two or three audio-recorded MI sessions each (32 sessions in total). The nurses were told that the sessions should last for at least ten minutes, preferably 20 min, and that at least one target behavior should be established for each session. The target behaviors are presented in Table 2. For one recorded session, the target behavior could not be established.

**Table 2:** Target behaviors for the recorded sessions

Target behaviors	Number^a^
Quit smoking.	7
Make lifestyle changes to improve health.	6
Gain control over blood sugar (with diet, exercise and medication).	2
Lose weight. Improve diet and exercise habits.	2
Maintain control over blood sugar (medication, complications, diet)	1
Maintain lifestyle improvements to gain control over blood sugar/metabolic syndrome including ordination adherence.	1
Quit smoking. Improve diet and exercise habits.	1
Lower blood pressure through lifestyle changes.	1
Improve diet and exercise habits. Explore origin of cough.	1
Maintain lifestyle improvements.	1
Join quit smoking group.	1
Begin asthma treatment.	1
Adherence to medication/treatment. Maintain non-smoking.	1
Follow eczema treatment recommendations.	1
Establish sleep routines and begin social training.	1
Establish sleep routines.	1
Actively contribute to language development and eat less sweets.	1
Seek help for depression.	1

^a^One target behavior is missing

#### Questionnaires

After each session, the nurses completed a questionnaire in which they rated their own MI performance. One part of the questionnaire contained five statements regarding the five global dimensions in the MITI: “In this session, I made an effort to explore the patient’s own thoughts about behavior change” (=Evocation), “In this session, I showed my awareness that the patient possesses the knowledge needed to achieve change” (=Collaboration), “In this session, I made an effort to promote the patient’s experience of having control and choice” (=Autonomy/Support), “In this session, I made an effort to focus on change” (=Direction) and “In this session, I made an effort to understand the patient’s perspective and feelings” (=Empathy). The participating nurses rated their performance on a 5-point Likert scale (1 = to a small extent – 5 = to a great extent). The second part of the questionnaire contained four questions regarding behavior counts in the MITI. The first was “If you consider the statements you made in this session, what do you think the relative proportions of reflections and questions were?” The response alternatives were: more questions than reflections, as many questions as reflections, or more reflections than questions. The other three questions were worded similarly, but concerned the relative proportions of statements that were MI adherent vs. MI Non-adherent, open vs. closed questions and complex vs. simple reflections (in each pair, the first-mentioned type reflects better MI performance). The response alternatives for these three questions mirrored those for questions vs. reflections.

Background information on the nurses and patients was also collected.

### Procedure

After ethical approval, permission for the study was obtained from the local authorities and the directors of primary care in the respective county council districts (Division Primary care at the county council district of Gävleborg and Primary care administration at the county council of Uppsala). Both contacted county councils agreed to participate in the study. The directors’ central administration provided the names of all nurse managers in the county council, who then were contacted to obtain the names of nurses who worked with MI, had received training in MI and/or worked with different specialist tasks. The intention was to receive names of all primary care nurses who met the criteria but some may have been missed because there were no current lists. The first author contacted the nurses by telephone and asked about their training in and use of MI. Nurses who met the inclusion criteria were invited to participate and were first verbally informed about the study. All nurses who chose to participate were also given written information about the study and asked to sign a consent form. The patients received oral and written information about the study at their visit to the nurse/primary care center, and their written consent to make a recording was obtained. The first author provided the recording equipment.

### Data analysis

The MITI, version 3.1.1 [19], was used to analyze the recorded MI sessions. The MITI has been tested for validity and reliability [42, 43]. To establish good inter-rater reliability, the MITI analysis was carried out by two coders at the Motivational Interviewing Coding Laboratory (MIC lab) at Karolinska Institute in Stockholm, Sweden, who work with the MITI. The MITI involves two different coding procedures: overall estimations of global dimensions (*global score*) and frequency calculations of specific verbal behaviors (*behavior counts*). Both procedures are performed at the same time during a single review of the tape. The results from the two coding procedures (global scores and behavior counts) are summarized to form an index (*summary score*) intended to assess the MI provider’s beginning proficiency/competence [19].

#### Global score

The overall estimations (global scores) comprise global estimations of five dimensions: Empathy, MI Spirit (containing the sub-dimensions Evocation, Collaboration and Autonomy Support) and Direction. The global scores are intended to capture the coders’ overall impression of the degree to which the provider (in this case the nurse) meets the criteria for each dimension. The overall impression of each dimension is rated on a 5-point Likert scale from 1(low) to 5 (high). The coders’ ratings are to characterize the entire part of the session that was coded [19].

#### Behavior counts

Here, the coder counts the number of occasions on which a specific verbal behavior occurs, during the part of the session that was coded. The specific verbal behaviors included in the count are: Giving Information, MI Adherent statements, MI Non-Adherent statements, Questions (Closed vs. Open) and Reflections (Simple vs. Complex). MI adherent statements entail asking for permission before giving information and advice, emphasizing the patient’s control, affirming and supporting the patient. MI Non-Adherent statements entail giving advice and information without permission, confronting the patient and giving orders [19].

#### Summary score

The summary scores serve as outcome measures for determining the MI provider’s MI competence. The summary score (calculation formulas are presented in parentheses) includes the global rating of MI spirit (evocation + collaboration + autonomy support/3), percent Complex reflections (complex reflections/all reflections), percent Open questions (open questions/all questions), reflection-to-question ratio (all reflections/all questions) and percent MI Adherent (MI Adherent/MI Adherent + MI Non Adherent statements). Thresholds for beginning proficiency and competency are recommended for each index [19].

Intra-class correlations (ICCs) were used to estimate the inter-rater reliability of the MITI coding in the present study, based on 14 (44 %) randomly selected recorded sessions [41]. When calculating and interpreting ICCs, a two-way mixed model with absolute agreement and single measures were employed in IBM SPSS Statistics, version 20. ICCs for the global scores ranged from 0.14 to 0.55 and for the behavior counts from 0.66 to 0.96, and are presented in Table 3. Guidelines for interpretation of ICC suggest that scores <0.4 represent poor agreement, 0.41-0.59 fair, 0.6-0.74 good and 0.75-1 excellent agreement [44]. The first author participated in the coding of 16 sessions to become familiar with the MITI coding process. Interpretation of the coding results was done by the first author and discussed with the other authors.

**Table 3:** Intra-class correlation coefficients (ICCs) for MITI variables

MITI variables	ICC	(95 % Cl)
Empathy	0.42	(-0.12-0.77)
Evocation	0.14	(-0.42-0.62)
Collaboration	0.49	(0.00-0.80)
Autonomy/Support	0.34	(-0.25-0.73)
Direction	0.55	(0.08-0.82)
Giving information	0.88	(0.66-0.96)
MI adherent	0.66	(0.02-0.89)
MI non-adherent	0.72	(0.33-0.90)
Closed questions	0.96	(0.88-0.99)
Open questions	0.95	(0.87-0.99)
Simple reflections	0.69	(0.29-0.89)
Complex reflections	0.83	(0.57-0.94)

*Note:* MITI = Motivational Interviewing Treatment Integrity Code

The nurses’ ratings of their own MI performance were analyzed to determine which MITI variables they rated themselves highest and lowest on. Their self-ratings were also compared with their coded scores and counts on the MITI to determine how well their perceptions of their performance accorded with their actual MITI scores/counts. This analysis was carried out by counting the ratings on every question, both for all nurses together and for each nurse separately. When comparing with their actual scores and counts, the steps between their own ratings and scores/counts of actual performance were also counted. Descriptive statistics were computed for the nurses’ and patients’ background variables and are described using ranges and means for continuous variables and frequencies and percentages for categorical variables.

### Ethical considerations

The study was approved by the Regional Ethical Review Board at the University of Uppsala (registration no. 2010/153). The principles of research ethics stipulated in the Helsinki Declaration [45] and Swedish Codes of Statues [46] have been followed. Written as well as oral information about the study was given to all participants. All participants were informed that the study was voluntary and that they could withdraw at any time without giving a reason. All data have been treated confidentially; the results contain no information that can reveal the participants’ identity.

## Results

### Characteristics of the nurses’ training in and use of MI

**Fig. 2:**
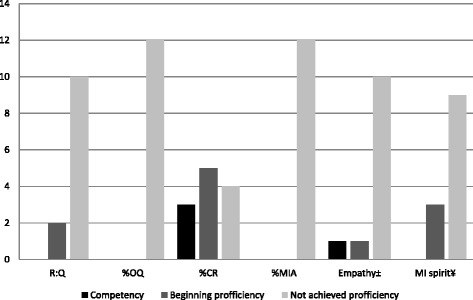
Number^#^ of primary care nurses (n = 12) who achieved beginning proficiency/competency^*^ or not in MI. *Notes:* MI = Motivational Interviewing, R:Q = Reflection-to-question ratio, %OQ = Percent open questions, %CR = Percent complex reflections, %MiA = Percent MI adherent. # Mean value of their conversations. *According to the Motivational Interviewing Treatment Integrity 3.1.1, Summary Score – Thresholds. ± Rating of empathy. ¥ Rating of MIspirit (Evocation, Collaboration and Autonomy)

Out of 200 possible nurses, 152 declined, 32 dropped out and 14 finally participated in the study, whereof two were excluded, as shown in Fig. 2. The participating nurses had used MI for 2-11 years (mean 5.6 years) and to different degrees. Five of the nurses reported using MI 2-6 times a week and five reporting using it ≥100 times a week. Two of the nurses were unsure. More information about the nurses’ training is presented in Table 4. The nurses are numbered and sorted in the table by their summary score on the MITI, from Nurse 1 (highest scores) to Nurse 12 (lowest scores). Their summary scores are shown in Table 7.

**Table 4:** Characteristics of the primary care nurses’ training in motivational interviewing (MI)

Nurse	Years since MI training	Days of MI training	Supervision	Feedback	Who provided the feedback^c^	How they received the feedback^d^
1	8	10^b^	No	Yes	A	L, A,
2	5	3	Yes	Yes	B	V, P
3	2	2^b^	Yes	Yes	B, E	A, V, G, P, W
4	6	3	No	No	-	-
5	^a^	2^b^	No	No	-	-
6	4	7^b^	Yes	Yes	B, C	L, O, V, G
7	7	2	No	No	-	-
8	12	7^b^	Yes	Yes	B	L, A
9	5	3	Yes	Yes	C	O
10	12	2.5	Yes	Yes	D	V, P
11	10	10	No	No	-	-
12	9	3	No	No	-	-

^a^no answer was given, ^b^Additional MI training not from the county council, e.g. Short course in MI, Asthma/COPD university education/course and university education/course in Disease Preventive methods, ^c^A = coding lab, B = MI educator, C = colleague, D = patient, E = supervisor, ^d^L = listen, A = analysis/coding, O = observation, V = verbally, G = in a group, P = in private, W = in writing

### Scores on global dimensions of the MITI vs. self-ratings of MI performance

As shown in Table 5, the nurses received the highest scores on Direction and the lowest on Collaboration and Evocation. They self-rated high on all five dimensions, showing great overestimation of their performance compared with their MITI scores on four dimensions, whereas their self-ratings on Direction were consistent with their scores. The nurses with the highest MITI scores had the most accurate self-ratings of MI performance, and those with the lowest MITI scores had the least accurate self-ratings.

**Table 5:** Global scores and self-ratings of primary care nurses’ motivational interviewing (MI)

		Global score^a^ and Self-ratings^b^				
Primary care nurses		Evocation	Collaboration	Autonomy/Support	Direction	Empathy
	Session	I/II/III	I/II/III	I/II/III	I/II/III	I/II/III
Nurse 1	Global score	3/4/3	3/4/4	3/3/4	4/4/4	4/4/4
	Self-ratings	3/4/4	3/4/4	4/4/4	3/4/4	4/4/3
Nurse 2	Global score	3/3/3	3/3/3	3/3/4	4/5/5	4/4/3
	Self-ratings	4/4/4	4/4/4	3/4/4	4/4/4	4/4/4
Nurse 3	Global score	2/3/^c^	2/3/^c^	3/2/^c^	5/5/^c^	4/4/^c^
	Self-ratings	3/^d^/^c^	2/^d^/^c^	3/^d^/^c^	4/^d^/^c^	3/^d^/^c^
Nurse 4	Global score	3/2/2	2/2/2	3/2/2	4/4/5	3/3/4
	Self-ratings	4/5/5	5/4/5	5/4/4	3/5/5	4/4/5
Nurse 5	Global score	3/2/1	3/2/2	4/3/1	5/5/4	4/3/2
	Self-ratings	5/4/3	4/4/3	5/4/4	5/4/4	5/4/4
Nurse 6	Global score	2/4/^c^	2/4/^c^	2/3/^c^	5/5/^c^	3/4/^c^
	Self-ratings	4/4/^c^	4/4/^c^	4/3/^c^	3/4/^c^	4/4/^c^
Nurse 7	Global score	^e^/2/1	2/2/2	2/2/2	^e^/2/3	3/2/3
	Self-ratings	4/4/4	4/4/5	3/3/4	4/4/5	4/4/4
Nurse 8	Global score	1/3/2	3/2/2	4/3/3	1/5/5	3/3/3
	Self-ratings	^d^/4/^c^	^d^/3/^c^	^d^/4/4	^d^/2/2	^d^/2/4
Nurse 9	Global score	2/3/2	3/4/1	3/3/2	2/3/3	3/3/2
	Self-ratings	3/4/4	5/4/4	5/3/4	3/3/4	3/4/5
Nurse 10	Global score	1/1/2	1/1/2	1/2/2	5/5/3	2/2/3
	Self-ratings	4/3/3	4/4/5	4/3/4	4/3/4	4/4/4
Nurse 11	Global score	1/2/^c^	1/1/^c^	1/1/^c^	5/5/^c^	1/2/^c^
	Self-ratings	5/5/^c^	5/5/^c^	5/5/^c^	5/5/^c^	5/5/^c^
Nurse 12	Global score	1/1/^c^	2/1/^c^	3/2/^c^	2/4/^c^	2/2/^c^
	Self-ratings	4/3/^c^	4/4/^c^	4/4/^c^	3/3/^c^	4/3/^c^
Summary of scores and self-ratings		Number of Sessions/Number of Nurses				
Global scores	*High score (4-5)*	2/2	4/3	4/4	23/10	10/6
	*Low score (1-2)*	19/10	20/10	15/9	4/4	9/6
Self-ratings	*High rate (4-5)*	21/11	25/10	23/11	20/10	25/11
	*Low rate (1-2)*	0	1/1	0	2/1	1/1
Overestimates^f^	*(+2 steps)*	10/6	7/7	9/6	2/1	5/4
	*(+3-4 steps)*	5/7	9/6	4/3	0	3/3
Underestimates^f^	*(-2-4 steps)*	0	0	0	4/3	0

^a^Global score on the nurses MI according to the Motivational Interviewing Treatment Integrity Code (MITI 3.1.1) Likert scale; 1 (low) – 5 (high)

^b^Participating nurses’ self-ratings of MI performance on a Likert scale: 1(to a small extent) – 5 (to a great extent)

^c^Missing session

^d^Missing answer

^e^Not possible to estimate because no target behavior was specified

^f^Self-ratings compared with scores

### MITI counts of specific behaviors vs. self-ratings of performance

The number of behavior counts is displayed in Table 6. There is variation within individual nurses’ own sessions as well as between nurses. Variation within own sessions is marked as bold numbers in Table 6, which shows that one nurse could have one count between two of her sessions, while another could have 45. The variation between nurses reveals a pattern, such that nurses with lower summary scores show the most variation within their sessions and those with higher summary scores show less variation. Looking at the nurses’ self-ratings (only presented as sums at the bottom of Table 6), they reported using more Simple reflection than Complex, more Open questions than Closed and more MI adherent than Non-adherent behavior than was indicated by their coded sessions. In relation to their actual counts, they overestimated themselves most on use of Open questions and underestimated themselves most on use of Complex reflections. (Under- and over-estimations are summarized at the bottom of Table 6 and marked with different symbols in the table). The behavior Giving information was not rated.

**Table 6:** Behavior counts and summary of self-ratings of performance in motivational interviewing (MI) for primary care nurses

			Behavior counts^a^						
Primary care nurses	Length of coded part of the session^b^ (minutes)	Length of session (minutes)	Giving information	MI Adherent (MiA)	MI Non-adherent (MiNa)	Closed questions (cq)	Open questions (oq)	Simple reflections (sr)	Complex reflections (cr)
Session	I/II/III	I/II/III	I/II/III	I/II/III	I/II/III	I/II/III	I/II/III	I/II/III	I/II/III
Nurse 1	20/20/20	51/51/54	18/20/22	2^d^/0/5^d^	0/0/1	24^e^/15/15	19/21/13	***24/33/20***	15/21/15
Nurse 2	20/20/20	54/50/48	15/18/14	5^e^/1/2^d^	0/0/0	16^e^/20^e^/12^e^	9/8/7	13^e^/6^e^/4	6/5/5
Nurse 3	20/20/^c^	24/42/^c^	15/15/^c^	7^d^/1/^c^	6/2/^c^	12/14/^c^	3/3/^c^	6^d^/10/^c^	13/15/^c^
Nurse 4	14/20/20	21/41/84	10/17/15	1/3^e^/1^e^	1/7/6	14^e^/15^e^/9	8/7/10	8^d^/10^d^/3^d^	12/12/8
Nurse 5	18/20/20	30/44/40	21/22/21	4/5^d^/1^e^	2/4/6	15^e^/21^e^/15	8/17/12	10^d^/7/8^e^	***15/6/5***
Nurse 6	17/20/^c^	20/26/^c^	**4/28/** ^**c**^	1^e^/2/^c^	3/0/^c^	***17*** ^e^ ***/7*** ^e^ ***/*** ^**c**^	5/8/^c^	10/9/^c^	3/2/^c^
Nurse 7	11/11/19	13/17/30	***14/10/30***	2^e^/1^e^/0	4/8/7	20/14^e^/11	8/8/2	2^d^/2^d^/2	11/4/7
Nurse 8	14/20/20	21/22/21	**22/35/46**	3/5^d^/2^e^	3/4/5	**14/49/33** ^e^	3/6/5	7/13/7^d^	***5/12/17***
Nurse 9	18/16/18	24/22/23	***17/23/34***	8/10^d^/5	3/2/7	***12*** ^e^ ***/22*** ^e^ ***/21*** ^e^	***9/15/3***	4^d^/3/8	8/3/1
Nurse 10	20/20/20	36/23/23	***0/18/15***	1^e^/2^e^/1^e^	9/6/5	**8/30** ^*e*^ **/20** ^*e*^	7/5/6	***8*** ^d^ ***/18/7***	5/7/8
Nurse 11	20/20/^c^	30/31/^c^	21/14/^c^	0^e^/2^e^/^c^	***17/5/*** ^**c**^	***20*** ^e^ ***/38*** ^e^ ***/*** ^**c**^	7/5/^c^	7/12/^c^	2/2/^c^
Nurse 12	20/20/^c^	37/29/^c^	**3/48/** ^**c**^	3/5^e^/^c^	2/9/^c^	***16*** ^e^ ***/26*** ^e^ ***/*** ^**c**^	8/5/^c^	3^e^/3^e^/^c^	0/0/^c^
Summary of self-ratings of performance			Number of Sessions/Number of Nurses						
Self-ratings		***More MiA/oq/cr*** *than MiNa/cq/sr*		9/6		11/7		2/2	
		***As many*** *MiA/oq/cr as MiNa/cq/sr*		13/9		13/9		11/6	
		***More MiNa/cq/sr*** *than MiA/oq/cr*		6/5		5/5		16/10	
Self-ratings compared with counts		*Overestimates*		14/9		21/11		5/3	
		*Underestimates*		7/6		0		10/7	

*Notes:* Bold numbers = variation in counts ≥ 20, Bold and italic numbers = variation in counts ≥ 10-19 (Variation in counts between the nurse’s own sessions)

^a^Behavior count on nurses’ MI according to the Motivational Interviewing Treatment Integrity Code (MITI 3.1.1)

^b^In MITI coding of MI sessions, 20 min (at least 10 min) from the beginning of the session (if/when there is a conversation) is coded

^c^Missing session

^d^Underestimates (Nurses self-ratings compared with counts)

^e^Overestimates (Nurses self-ratings compared with counts)

### Proficiency/competency in MI as stipulated by MITI thresholds

The summary score results are presented in Table 7 and show that none of the nurses achieved beginning proficiency on every variable/summary score or for all sessions. Two nurses did not achieve beginning proficiency on any summary score. The nurse with the best performance exceeded the threshold for competency on four variables and for beginning proficiency on six, across all her sessions. The same nurse also achieved the most beginning proficiency/competency in one and the same session, on four of the six variables. The summary score “percent complex reflection” was the one on which most nurses achieved beginning proficiency/competency, followed by “empathy,” and the summary scores on which the fewest achieved beginning proficiency/competency were “percent open question” and “MI spirit rating”. The mean scores for each nurse’s beginning proficiency/competency across all sessions are presented in Fig. 2.

**Table 7:** Primary care nurses’ summary scores/proficiency in motivational interviewing (MI)

		Summary scores^a^					
Primary care nurses		Reflections-to-questions ratio	Percent open questions	Percent complex reflections	Percent MI adherent	Empathy	MI spirit rating^b^
*Thresholds:*	*Beginning proficiency*	≥1	≥ 50 %	≥ 40 %,	≥ 90 %	**≥** 3.5	≥ 3.5
	*Competency*	≥ 2	≥ 70 %	≥ 50 %	≥ 100 %	≥ 4	≥ 4
	Session	I/II/III	I/II/III	I/II/III	I/II/III	I/II/III	I/II/III
Nurse 1		0.9/***1.5***/***1.3***	44/***58***/46	38/39/***43***	**100/**0/83	**4**/**4**/**4**	3/***3.7***/***3.7***
Nurse 2		0.8/0.4/0.5	36/29/37	32/***45***/**56**	**100**/**100**/**100**	**4**/**4**/3	3/3/3.3
Nurse 3		***1.3***/***1.5***/^c^	20/18/^c^	**68**/**60**/^c^	54/33/^c^	**4**/**4**/^c^	2.3/2.7/^c^
Nurse 4		0.9/***1.0***/0.6	36/32/***53***	**60**/**55**/**73**	50/30/14	3/3/**4**	2.7/2/2
Nurse 5		***1.1***/0.3/0.5	35/45/44	**60**/***46***/38	67/56/14	**4**/3/2	3.3/2.3/1.3
Nurse 6		0.6/0.7/^c^	2/***53***/^c^	23/18/^c^	25/**100**/^c^	3/**4**/^c^	2/***3.7***/^c^
Nurse 7		0.5/0.3/0.7	29/36/15	**85**/**67**/**78**	33/11/0	3/2/3	1.3/2/1.7
Nurse 8		0.7/0.4/0.6	18/11/13	***42***/***48***/**71**	50/56/29	3/3/3	2.7/2.7/2.3
Nurse 9		0.6/0.2/0.4	43/41/13	**67**/**50**/11	73/83/42	3/3/2	2.7/3.3/1.7
Nurse 10		0.9/0.7/0.6	47/14/23	38/28/**53**	10/25/17	2/2/3	1/1.3/2
Nurse 11		0.3/0.3/^c^	26/12/^c^	22/14/^c^	0/29/^c^	1/2/^c^	1/1.3/^c^
Nurse 12		0.1/0.1/^c^	33/16/^c^	0/0/^c^	60/36/^c^	2/2/^c^	2/1.3/^c^

*Notes:* Boldface numbers indicate achieved beginning proficiency or competency. Boldface and italics = Beginning proficiency and just Bold = Competency

^a^Summary scores on the nurses MI according to the Motivational Interviewing Treatment Integrity Code (MITI 3.1.1)

^b^MI spirit rating the score of Evocation + Collaboration + Autonomy Support/3

^c^Missing session

## Discussion

The main finding of the present study was that none of the nurses achieved beginning proficiency in all parts of one MI session and that two nurses did not achieve beginning proficiency in any parts or sessions. Making more complex than simple reflections was the specific MI verbal behavior/summary score on which most nurses (nine nurses in 19 sessions) exceeded the threshold for beginning proficiency. Four nurses only achieved beginning proficiency for this specific behavior. The nurses self-rated being poor at reflection, and especially at complex reflection, and they self-rated being good at open questions. However, their summary scores show that only three nurses (in one session each) achieved beginning proficiency in asking open questions.

Comparing nurses’ self-ratings of performance on questions and reflections compared with their MITI scores indicates that it is important to evaluate skills and get feedback on one’s own MI performance if one is to improve. The nurses’ reports on their MI training also indicate that those with extensive MI training and/or who have received feedback are also those with higher MITI scores, with one exception (Nurse 4). This may to some extent be explained by the fact that Nurse 4 worked with a colleague who used MI and got feedback from another colleague who was an MI instructor; another explanation is that individuals learn MI differently. Previous reviews have shown, however, that short MI training courses do improve MI skills [12, 37, 47], but that extended contact including feedback or supervision results in more skill retention [37, 47]. Recurrent evaluation using assessment tools such as the MITI is also recommended to promote continuing development of MI skills [20, 26].

The fact that none of the nurses actually achieved beginning proficiency in all parts of any session may indicate that they have received too little training, feedback, supervision and/or support. In a survey study [39] conducted in the same setting, Swedish primary care nurses reported lack of knowledge, professional follow-up and support as primary obstacles to their use of MI [39]. Nurses have also described how difficult it is to change old habits and relearn new methods such as MI, and that it is easy to revert to old conversation techniques when one is tired or has a heavy workload. Therefore, it was concluded that support from colleagues and management as well as more practice and feedback are required [25, 48]. Without proper guidance, medically trained personnel may assume an approach that is authoritarian, confrontational, forceful or guilt-inducing. There is evidence suggesting that such attitudes not only limit progress, but also correlate with negative behavioral and clinical outcomes, such as resistance to change [49]. The fact that MI is the opposite of the former confrontational, advice-giving conversational approach may also explain why so few of the nurses in the present study achieved beginning proficiency/competency and so many showed so much variation in MI behavior between their own sessions.

The nurses in the present study scored low on the MITI measure MI spirit (Evocation, Collaboration and Autonomy/Support). One challenge in many conversations in primary care is the need to simultaneously focus on many different goals (e.g., quit smoking, increase physical activity, change diet) and to motivate the patient to maintain certain behaviors while changing others, as well as the need to provide the information required by various recommendation/guidelines [20]. The nurses also had low proportions of open questions and reflections, and they gave a great deal of information/advice without permission. According to the developers of MI [3], open questions play a key role in eliciting motivation and supporting collaboration. Reflective listening, showing true empathy, is an essential skill in MI and is used to keep the patient exploring and considering his/her options. Asking for permission before giving advice and information is also important in supporting autonomy and avoiding the confronting expert role, which may create resistance to change. Previous studies [15, 49, 50] have also shown that behavior consistent with MI (open questions, reflections, advice with permission, affirmation, emphasizing control and support) is associated with patients’ “change talk.” The nurses in the present study estimated that they used more open than closed question, which was also shown in a study of nurses in diabetes care [25]. Such beliefs may further obstruct nurses’ development of MI skills, because they think they are better at some aspects of the method than they actually are. MI providers need to be aware of their performance if they are to progress and to be able to help patients become motivated to change. Another phenomenon we observed from the audio-recorded sessions was that the nurses at times read their questions from a self-made checklist of various lifestyle changes. Such questions can never be counted as open and were automatically coded as closed, which is another point for MI providers to keep in mind.

### Strengths and limitations

According to guidelines [44] for interpretation of ICCs, the ICCs of the global scores Evocation and Autonomy/Support were poor in the present study, which means that these variables have low reliability. The implication of this limitation has been and must be considered when interpreting the findings, and conclusions should be made with caution. This ICC result can be explained by the small sample of sessions or/and the complex setting of primary care in which the conversations took place, as discussed above. Other possible explanations are the fact that the sessions may involve many target behaviors for the nurses to focus on, sometimes in a very short time, and the fact that nurses do not always intend to motivate for change but only for maintenance, which MI is not intended to address in the first place [3, 20]. Further, an entire session is rarely devoted to motivation and the need for change only, but may also contain various examinations. These elements may have made it unclear as to whether the nurses were, for example, evoking as well as made it difficult for the coders to carry out their ratings. Moreover, the coders are used to rating conversations that focus on one target, such as quitting smoking or drinking, which may have made the coding more difficult. This is also confirmed in a previous study on Swedish child health services in primary care [20].

Choosing two county council districts was considered sufficient for obtaining variation in the sample and a sample that represented conditions in Sweden, provided the intended number of participants was finally included in the sample. It is possible, of course, that inclusion of more counties would have generated more knowledge. Because many nurses declined to participate and dropped out, we do not know whether or not the participating nurses are representative of all primary care nurses and, thus, the results cannot be generalized. We can only speculate that the participating nurses were most likely those interested in using MI and in improving their MI performance. It is also possible that the participating nurses were more comfortable with the method and used it more often. Another potential limitation is the fact that the sessions were self-selected by the nurses and may therefore provide a biased view of the MI skill level of nurses in primary care. The nurses who participated varied in terms of the MI training and supervision they had received, and this does reflect the actual situation for primary care nurses. In the present study, we have therefore described their MI performance irrespective of their previous training and supervision.

Many nurses declined to participate due to time pressure, which may be seen as lack of organizational support. At primary care centers where management was supportive of participation, the nurses were more willing to participate. Another reason for declining was reluctance to record their sessions for performance assessment, which may be interpreted as reflecting their uncertainty about their use of MI, thus further stressing the need for more support and training. Comparable concerns about lack of support from the organization were also noted in a previous study in a similar setting [20]. Even though the number of participants was small, the purpose of the present study was to describe performance and self-ratings of performance as it naturally occurs, and the 32 sessions studied provided a wide range of interesting information about patterns within and between the nurses and their sessions. Despite its limitations, the present study contributes to our knowledge of MI sessions in primary care nurses’ real work settings and with their real prerequisites regarding training and use.

## Conclusions

Primary care nurses did not achieve beginning proficiency in all aspects of MI in their recorded sessions targeting lifestyle change. This indicates a need for more training, feedback and supervision in clinical practice with MI to promote improvement. It is also important to assess MI performance before MI outcome studies are carried out to ensure that what is supposed to be measured is actually being measured as well as to help MI providers understand their own performance, with a view to improving their MI use and skills.

## Abbreviations

MI: Motivational interviewing
MITI: Motivational interviewing integrity code
